# Yoga and its effect on sperm genomic integrity, gene expression, telomere length and perceived quality of life in early pregnancy loss

**DOI:** 10.1038/s41598-024-62380-w

**Published:** 2024-05-22

**Authors:** Vidhu Dhawan, Neena Malhotra, Neeta Singh, Vatsla Dadhwal, Rima Dada

**Affiliations:** 1 Department of Anatomy, Laboratory of Molecular Reproduction and Genetics, All India Institute of Medical Sciences, New Delhi, 110029 India; 2 Department of Obstetrics and Gynecology, All India Institute of Medical Sciences, New Delhi, India

**Keywords:** Yoga, Meditation, Sperm transcripts, Oxidative stress, DNA damage, Telomere, Gene expression, Genetics, Molecular biology, Medical research

## Abstract

Achieving successful pregnancy outcomes is a delicate interplay between the maternal and the fetal counterparts. Paternal factors play a critical role in health and disease of offspring. Early pregnancy loss (EPL) is a psychologically devastating condition affecting the quality of life (QOL). Thus, it needs to be managed by a mind body integrated approach like yoga.The prospective single arm exploratory studyincluded male partners of couples experiencing recurrent pregnancy loss (RPL, n = 30), and recurrent implantation failure (RIF, n = 30) and semen samples wereassessed at the beginning and completion of yoga (6 weeks) (WHO 2010).

A significant increase in the sperm concentration, motility, decrease in seminal ROS, DFI and increase in relative sperm telomere length was found at the end of yoga. The relative expression of genes critical for early embryonic developmentnormalized towards the levels of controls. WHOQOL-BREF questionnaire scores to assess QOL also showed improvement.

Integration of regular practice yoga into our lifestyle may help in improving seminal redox status, genomic integrity, telomere length, normalizing gene expression and QOL, highlighting the need to use an integrated, holistic approach in management of such cases. This is pertinent for decreasing the transmission of mutation and epimutation load to the developing embryo, improving pregnancy outcomes and decreasing genetic and epigenetic disease burden in the next generation.

## Introduction

A significant increase in complex lifestyle diseases has been witnessed in the last decade with a dramatic impact on the reproductive potential. Paternal factors have been cited as an important contributor in the normal embryonic development and associated as a causal factor for early pregnancy loss (EPL). Paternal factors play a role in each stage of embryo development from fertilization, with early and late paternal effects and implantation. Integration of yoga a mind body energy medicine for the management of complex chronic lifestyle conditions as an adjunct in the management of infertility and EPL has been documented and is highly effective in these psychosomatic disorders. Advancing paternal age at childbirth due to various social, psychological, career advancements, delayed age at marriage, increase in life expectancy are among a few factors to initiate family at a later age. The delayed parenthood results in poor reproductive outcome due to cumulative damage to germ cell especially sperm which has highly truncated DNA repair mechanism and minimal antioxidant capacity to combat oxidative insults due to various intrinsic and extrinsic factors and thus accumulates oxidative DNA damage, denovo germ line mutations and epimutations^1–4^.

Male reproductive fitness is not only essential for procreation but it also reflects his somatic fitness, and, impaired fertility may pose as a harbinger of medical diseases in men. Poor semen quality has been linked with an increasing risk of common chronic diseases^5,6^, morbidity and mortality^7,8^, thus highlighting their public health importance beyond fertility and reproduction.Semen parameters are negatively associated with stress and impacted by a decline in the levels of luteinizing hormone (LH) and testosterone, which further affects spermatogenesis and thus the sperm quality^9–11^.

The significant and positive Impact on the quality of life by yoga and meditation has witnessed an improvement in sperm functional parameters. The incorporation of yoga in psychosomatic conditions has been documented to play a significant role in decreasing severity of co-morbid depression & stress through the downregulation of the hypothalamic pituitary axis (HPA)and sympathetic nervous system, stress reduction and immune modulation^12–15^. This thus exerts beneficial effects on the regulation of reproductive hormones.

The transcriptionally inert sperm with minimal antioxidant defense is prone to be attacked by the overwhelming OS resulting in both nuclear and mitochondrial DNA, accelerated telomere shortening, accumulation of mutagenic bases and oxidative DNA adducts which induce mutations & epimutations.

We hypothesized that lowering of oxidative DNA damage in the spermatozoa with the adoption of yoga might improve the functional parameters in sperm, increase telomere length and modulate the gene expression in the male partners of couples who experienced recurrent pregnancy loss (RPL) and recurrent implantation failure (RIF). Yoga switches on the internal pharmacy. Recent evidences have established the link between the integration of yoga practices in the management of chronic complex lifestyle diseases and the health benefits it provides not only in management but also by preventing onset of these complex diseases and also by promoting health and wellness. With the similar intent, the primary aim of the current study is to assess the impact of 6-week yoga intervention on the sperm oxidative and molecular markers (gene expression profile) as well as quality of life in male partners of RPL and RIF patients.

## Results

### Participants flow and baseline age, BMI, seminal and oxidative markers in patient groups and controls

A total of 82 men who volunteered for the study and gave informed written consent, were initially recruited for this study, RPL (N = 42) and RIF (N = 40) groups. A total of 60 male partners were finally recruited in the study. The baseline levels demographic, seminal, oxidative parameters and telomere length are shown in Table 1. The patient groups and controls were age matched. The PM (%) and SC (million/ml) was found to be lower and significantly different (p < 0.0001****) in the patient groups as compared to the controls.

**Table 1:** Age, BMI, baseline seminal and oxidative parameters, and sperm telomere length in patients and controls.

Parameter	RPL	RIF	Control	p-Value		
				Overall	RPL vs Control	RIF vs Control
AGE (years)	33.6 ± 3.9	34.7 ± 3.6	32.4 ± 3.6	0.063	0.651	0.058
BMI (kg/m^2^)	25.2 ± 2.4	25.7 ± 1	24.3 ± 1.6	0.012*	0.161	0.010*
Volume (ml)	2.9 ± 1.0	3.1 ± 1.8	3.5 ± 1.02	0.169	0.196	0.636
pH	7.4 ± 0.16	7.5 ± 0.28	7.3 ± 0.24	0.019*	0.019*	0.135
LT (mins)	36.8 ± 14.7	39.1 ± 12.3	31.5 ± 5.6	0.035*	0.231	0.035*
PM (%)	49.5 ± 12.6	32.7 ± 12.5	64.3 ± 9.9	< 0.0001****	< 0.0001****	< 0.0001****
SC (million/ml)	38.9 (0.4, 145.5)	29.3(0.3, 60)	56.2 (23.6, 139.3)	0.0001***	**0.0321**	< 0.0001****
ROS						
(RLU/sec/million sperm)	39.9(3.5, 451.9)	44.9 (16.5, 382.6)	18.3 (4.83, 53.9)	< 0.0001****	< 0.0001****	< 0.0001****
DFI (%)	38.6 ± 5.6	39.4 ± 4.4	25.5 ± 6.21	< 0.0001****	< 0.001****	< 0.001****
T/S Ratio	0.520 ± 0.08	0.475 ± 0.08	0.655 ± 0.13	0.0001***	< 0.0001****	< 0.0001****

Values expressed as mean ± sd and median (minimum, maximum.) *p < 0.05 was considered significant. *p < 0.05, **p < 0.01, ***p < 0.001, ****p < 0.0001.

*BMI* Body mass index, *LT* Liquefaction time, *PM* Progressive motility, *SC* Sperm concentration, *ROS* Reactive oxygen species, *RLU* Relative light units, *DFI* DNA fragmentation index.

### Effect of yoga on semen parameters, seminal oxidative stress, DNA damage and sperm telomere length (STL)

**Figure 1:**
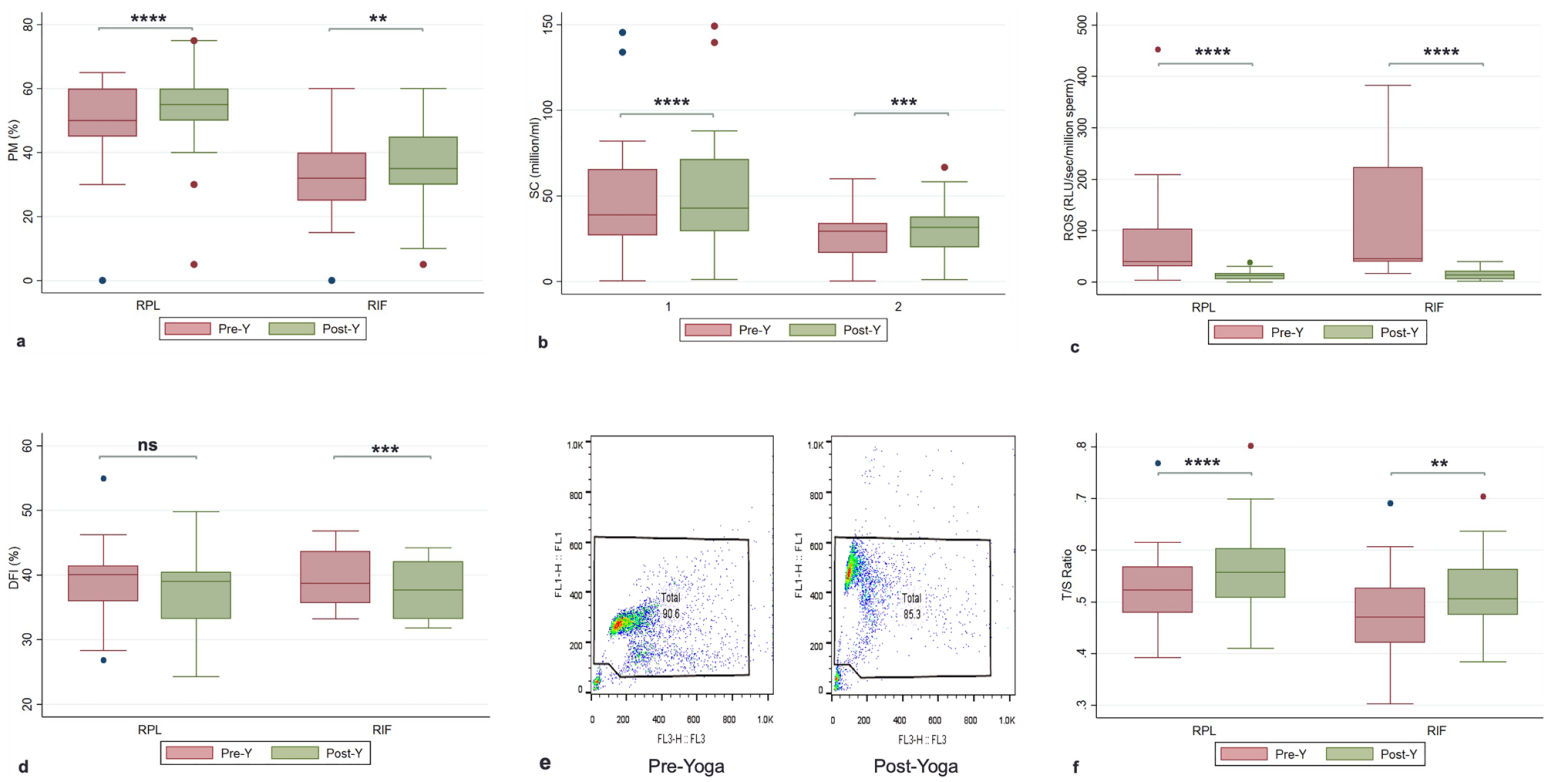
(**a**–**e**): Pre- and Post-Yoga levels of (**a**) Progressive motility (PM), (**b**) Sperm concentration (SC), (**c**) Reactive oxygen species (ROS), (**d**) DNA fragmentation index (DFI), (**e**) Pseudocolour dot plot cytograms of the semen samples of cases recruited for Yoga by SCSA at estimated at pre-yoga (day 0) and at post-yoga (6 weeks) FL3 on x-axis represents fragmented DNA, and FL1 on y-axis representative DNA showing the percentage DFI. Each dot in the cytogram represents a single spermatozoon with red and green fluorescence values. The debris at the bottom left corner was excluded from analysis, (**f**) Relative sperm telomere length (T/S Ratio). *p < 0.05, **p < 0.01, ***p < 0.001, ****p < 0.0001.

There was a significant increase in the mean PM in both RPL and RIF group (*p* < 0.0001**** and *p* = 0.0078** respectively, Table 2, Fig. 1a) and median SC in RPL and RIF group (*p* < 0.0001***, Table 2, Fig. 1b) from baseline to the post-yoga levels.The ROS and DFI levels were found to be significantly higher in RPL and RIF groups from controls (*p* < 0.0001****) (Table 2). The ROS levels showed significant decline from baseline to the end of 6 weeks of yoga (*p* < 0.0001****) (Table 2, Fig. 1c). The DFI also showed decline, but the change was found to be significant in RIF group (*p* = 0.0002***) and not in RPL group (*p* = 0.0714) (Table 2, Fig. 1d, 1e). The representative dot plot cytograms is shown in Fig. 1e.The baseline relative sperm telomere length (STL/ T/S ratio) was found to be significantly lower in patient groups than the controls (*p* < 0.001***) (Table 2) and showed a significant positive increase from the baseline levels in RPL (*p* < 0.001***) and RIF (*p* = 0.0088**) groups. (Table 2, Fig. 1f) and correlated negatively with ROS and DFI levels.

**Table 2:** Change in outcomes in the levels of variables following parametric distribution (PM, DFI and T/S ratio and change in outcomes in the levels of WHOQOL-BREF scores in the 4 domains) and variables following non-parametric distribution in RPL and RIF patients adopting Yoga program.

Variables following parametric distribution										
Group/outcome	Pre-yoga (Baseline)	Post-Y (6 weeks)	Change from baseline		Variance analysis/effects			Pearson’s correlation		
	(Mean ± sd)	(Mean ± sd)	Mean ± sd	95% CI	*p*-value (two-tailed)	t	$\eta_{p}^{2}$	r	95% CI	*p*-value
RPL group										
PM (%)	49.5 ± 12.6	54.5 ± 13.2	5.0 ± 3.4	3.78, 6.35	< 0.0001****	8.089	0.6929	0.9656	0.928, 0.984	< 0.0001****
DFI (%)	38.6 ± 5.6	37.6 ± 5.2	− 1.03 ± 3.15	− 2.21, 0.14	0.083	1.795	0.1000	0.8355	0.679, 0.919	< 0.0001****
T/S Ratio	0.520 ± 0.08	0.560 ± 0.090	0.040 ± 0.034	0.025, 0.055	< 0.0001****	5.748	0.6003	0.927	0.833, 0.969	< 0.0001****
RPL- WHOQOL scores										
Physical (D1)	46.5 ± 3.5	51.7 ± 3.1	5.2 ± 1.6	8.8, 14.15	< 0.001****	17.7	0.9156	0.893	0.786, 0.948	< 0.001****
Psychological (D2)	44.0 ± 4.0	51.1 ± 3.9	7.0 ± 1.4	6.5, 7.57	< 0.001***	25.9	0.958	0.931	0.86, 0.967	< 0.001***
Social (D3)	46.4 ± 5.3	52.8 ± 4.5	6.7 ± 3.9	5.3, 8.2	< 0.001***	9.3	0.752	0.690	0.439, 0.841	< 0.001****
Environmental (D4)	52.5 ± 3.3	52.8 ± 3.4	0.33 ± 0.75	0.05, 0.61	0.022*	2.4	0.1667	0.974	0.946, 0.988	< 0.001***
RIF group										
PM (%)	32.7 ± 12.5	35.6 ± 13.6	2.88 ± 5.26	0.92, 4.84	0.0055**	3.002	0.2371	0.9226	0.842, 0.963	< 0.0001****
DFI (%)	39.4 ± 4.4	37.9 ± 4.4	− 1.54 ± 1.6	− 2.15, − 0.943	< 0.001***	5.246	0.4869	0.9334	0.863, 0.968	< 0.0001****
T/S Ratio	0.475 ± 0.087	0.520 ± 0.074	0.043 ± 0.07	0.013, 0.073	0.0062**	3.015	0.2832	0.625	0.296, 0.821	0.0011**
RIF- WHOQOL Scores										
Physical (D1)	43.6 ± 3.1	49.0 ± 3.0	11.5 ± 6.4	5.4, 14.15	< 0.001***	15.2	0.8891	0.807	0.630, 0.904	< 0.0001****
Psychological (D2)	43.2 ± 3.1	50.4 ± 5.3	6.8 ± 4.4	5.1, 8.5	0.0009***	8.4	0.7084	0.545	0.230, 0.757	0.0018***
Social (D3)	47.1 ± 4.2	52.8 ± 4.7	5.7 ± 3.4	4.4, 6.9	< 0.0001****	9.13	0.742	0.714	0.476, 0.854	< 0.0001****
Environmental (D4)	53.6 ± 2.9	54.0 ± 2.7	0.4 ± 1.0	− 0.01, 0.81	0.057	2.0	0.2083	0.940	0.868, 0.973	< 0.0001****

Variables following non-parametric distribution										
Group/ outcome	Pre-yoga (Baseline)		Post-Y (6 weeks)		Median of differences	Variance analysis/effects		Spearman’s correlation		
	Median (min–max)	95% CI	Median (min–max)	95% CI		p-value (two-tailed)		r	95% CI	*p*-value
RPL Group										
SC (million/ml)	38.9 (0.4, 145.5)	29.8, 53.0	42.85 (1.2, 149.2)	31.6, 60.1	4.75	< 0.0001****		0.9748	0.946, 0.988	< 0.0001****
ROS (RLU/sec/10^6^ sperm)	39.95 (3.5, 451.9)	35.9, 85.02	12.6 (0.27, 37.8)	5.8, 14.2	-34.8	< 0.0001****		0.6245	0.331, 0.808	0.0002***
RIF group										
SC (million/ml)	29.3 (0.3, 60)	19.8, 31.8	31.7 (1.1, 66.7)	22.3, 35.6	2.7	0.0001***		0.890	0.789, 0.951	< 0.0001****
ROS (RLU/sec/10^6^ sperm)	44.9 (16.5, 382.6)	43.2, 164	14.2 (1.9, 39.6)	5.7, 21.3	-39.45	< 0.0001****		0.7409	0.511, 0.872	< 0.0001****

Values expressed as mean ± sd and median (minimum, maximum.) *p < 0.05 was considered significant. *p < 0.05, **p < 0.01, ***p < 0.001, ****p < 0.0001.

*PM* Progressive motility, *DFI* DNA fragmentation index, *SC* Sperm concentration, *ROS* Reactive oxygen species, *CI* Confidence interval, *t* t-test score, $\eta_{p}^{2}$ partial eta squared value representing the effect size, *r* correlation coefficient.

### Effect of yoga on sperm gene expression and QOL

**Figure 2:**
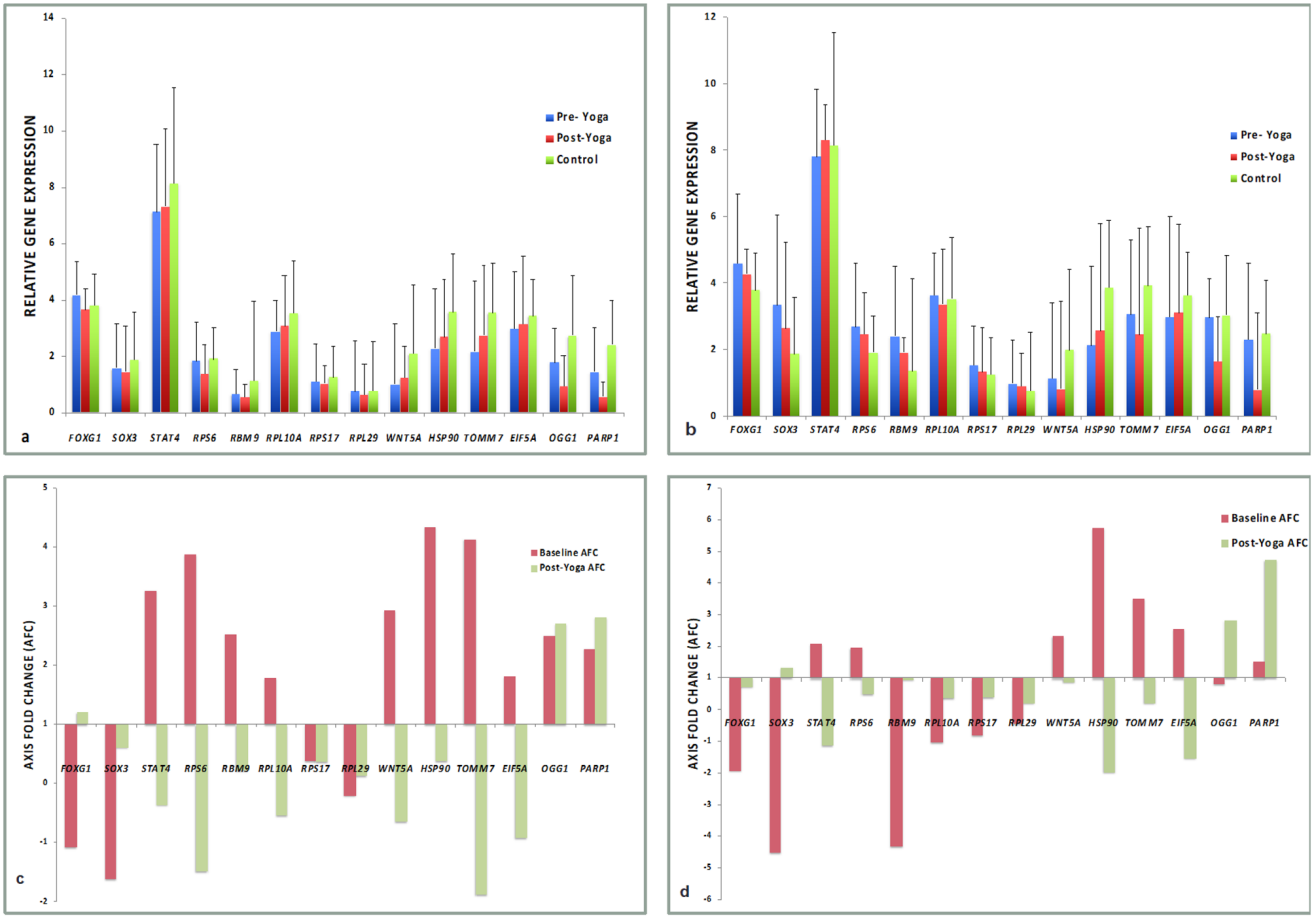
Relative gene expression (Average ΔCt) pre- and post-yoga (6 weeks) as well as controls in (**a**) RPL and (**b**) RIF patients; Axis fold change (AFC) in gene expression at baseline with respect to controls and post-yoga with respect to pre-yoga in (**c**) RPL and (**d**) RIF patients.

The relative expression of the genes at the end of yoga as compared to baseline was seen to normalize towards that of the control values in both RPL (Table 3, Fig. 2a) and RIF groups (Table 3, Fig. 2b). We found a significant difference in the baseline expression of *HSP90, TOMM7, OGG1 and PARP1* with respect to controls in RPL group. While the expression of *FOXG1, SOX3, RBM9,* and *HSP90,* showed significant difference as compared to controls in RIF group.

**Table 3:** Relative gene expression (Average ΔCt) and axis fold change (AFC) in gene expression at baseline with respect to controls and post-yoga levels with respect to pre-yoga levels in RPL and RIF patients.

GOI	Control	RPL						RIF					
	ΔCt Controls	ΔCt Pre- Yoga	ΔCt Post- Yoga	p-value	r value	Axis fold change		ΔCt Pre-Yoga	ΔCt Post-Yoga	p-value	r value	Axis fold change	
						Baseline	Post-yoga					Baseline	Post-yoga
*FOXG1*	3.8 ± 1.17	4.19 ± 1.17	3.65 ± 0.82	0.004**	**0.7353**	-1.09	1.21	4.6 ± 1.7	4.28 ± 1.35	0.171	**0.7484**	− 1.94	0.70
*SOX3*	1.88 ± 1.46	1.59 ± 1.58	1.45 ± 0.76	0.978	0.3901	− 1.62	0.61	3.35 ± 2.05	2.64 ± 1.32	0.0067**	**0.8553**	− 4.51	1.32
*STAT4*	8.14 ± 2.28	7.13 ± 2.4	7.32 ± 2.61	0.510	**0.7279**	3.26	− 0.36	7.8 ± 1.59	8.3 ± 1.32	0.127	**0.5876**	2.08	− 1.14
*RPS6*	1.92 ± 1.32	1.85 ± 1.22	1.38 ± 0.87	0.014*	**0.47**	3.87	− 1.49	2.7 ± 1.77	2.47 ± 1.55	0.255	**0.7392**	1.95	0.48
*RBM9*	1.15 ± 1.89	0.66 ± 0.86	0.57 ± 1.07	0.861	− 0.063	2.52	0.22	2.41 ± 1.35	1.92 ± 0.96	0.0083**	0.0108	− 4.32	0.94
*RPL10A*	3.54 ± 1.77	2.89 ± 1.11	3.07 ± 1.23	0.545	0.3844	1.78	− 0.54	3.64 ± 1.21	3.37 ± 0.71	0.946	0.3614	− 1.04	0.36
*RPS17*	1.26 ± 1.1	1.11 ± 1.34	1.04 ± 0.66	0.492	**0.7073**	0.38	0.37	1.53 ± 0.97	1.33 ± 081	0.151	0.2232	− 0.82	0.37
*RPL29*	0.77 ± 1.23	0.75 ± 1.8	0.63 ± 1.18	0.637	**0.6798**	-0.21	0.13	0.98 ± 0.96	0.9 ± 0.82	0.480	0.1669	− 0.45	0.21
*WNT5A*	2.1 ± 1.5	1.01 ± 2.15	1.24 ± 1.42	0.581	**0.6894**	2.93	-0.64	1.13 ± 2.13	0.82 ± 2.6	0.065	-0.207	2.33	0.86
*HSP90*	3.59 ± 1.58	2.28 ± 2.13	2.7 ± 1.82	0.252	0.3589	4.34	0.38	2.13 ± 2.49	2.56 ± 3.03	0.0003***	**0.5839**	5.73	− 1.97
*TOMM7*	3.56 ± 1.64	2.17 ± 2.5	2.75 ± 2.17	0.173	**0.5287**	4.12	− 1.88	3.06 ± 2.12	2.45 ± 2.39	0.106	0.1296	3.51	0.20
*EIF5A*	3.45 ± 1.32	2.99 ± 2.01	3.14 ± 1.75	0.736	0.1335	1.81	− 0.93	2.98 ± 2.53	3.11 ± 2.57	0.186	0.3376	2.54	− 1.55
*OGG1*	2.75 ± 2.15	1.78 ± 1.22	0.94 ± 1.98	0.073	0.262	2.52	2.7	2.97 ± 1.65	1.64 ± 2.04	0.523	0.2041	0.80	2.81
*PARP1*	2.4 ± 1.6	1.45 ± 1.6	0.56 ± 1.86	0.007**	**0.478**	2.27	2.8	2.3 ± 2.01	0.79 ± 2.09	0.945	0.2101	1.51	4.71

Values expressed as mean ± sd. *p < 0.05 was considered significant. *p < 0.05, **p < 0.01, ***p < 0.001, ****p < 0.0001.

GOI, Gene of interest; *FOXG1,* Forkhead box G1; *SOX3,* SRY (sex determining region Y)-box 3; STAT4, Signal transducer and activator of transcription 4); *RPS6,* Ribosomal protein S6); *RBM9,* RNA binding motif protein 9); *RPL10A,* Ribosomal protein L10A; *RPS17,* Ribosomal protein S17; *RPL29,* Ribosomal protein L29; *WNT5A,* Wnt Family Member 5A; *HSP90,* Heat shock protein 90; *TOMM7,* Translocase of outer mitochondrial membrane 7); *EIF5A,* Eukaryotic translation initiation factor 5A; *OGG1,* 8-oxoguanine DNA glycosylase; *PARP1,* Poly(ADP-ribose) polymerase 1; r, correlation coefficient.

The axis fold change in the gene expression at baseline levels in RPL and RIF group as compared to controls as well as the fold change post-yoga levels with respect to pre-yoga levels is shown in Table 3, Fig. 2(c) and (d ). The post-yoga expression of *FOXG1, RPS6* and *PARP1* varied significantly from the pre-yoga levels in RPL group, while the expression of *SOX3, RBM9* and *HSP90* showed significant difference from pre-yoga levels in RIF group. A significant correlation was seen between pre- and post-yoga expression of *FOXG1, STAT4, RPS17* (*p*- < 0.0001****), *RPS6* (p-0.017*), *RPL29* (0.0002***), *WNT5A* (0.0001***), *TOMM7* (0.006**), *PARP1* (0.015*) in RPL group and *FOXG1, SOX3, RPS6* (*p*- < 0.0001*), *STAT4,, HSP90* (0.001**) in RIF group***.*** The change in mean scores of QOL from baseline in WHOQOL-BREF scores in domains D1, D2 and D3 were found to be statistically significant, while a trend towards improvement was noted in domain D4 scores in both groups (Table 2). Significant positive correlation was observed between the pre-yoga and post-yoga levels (Table 2).

## Discussion

The present study attempted to comprehend the contributions of adoption of yoga and evaluate its impact on sperm function, oxidative seminal biomarkers (ROS, DFI), telomere length and gene expression. The study also compared the baseline characteristics in the RPL and RIF patient groups with the healthy fertile controls. We found a significant difference in the semen parameters (PM and SC), ROS, DFI and telomere length in patient groups with control group which improved after adoption and integration of yoga into their daily routine. The study attempted to highlight the changes in gene expression pattern, oxidative stress, DNA damage and telomere length with yoga and established a significant positive change in the quality of life in patients as established by the WHOQOL-BREF questionnaire.

Psychological stress and impending OS are one of the key components,which affect the sperm structure and function. OS affects the key sperm function i.e. the sperm motility, which was found to be lower in both RPL and RIF groups with respect to controls. Oxidative damage to sperm membrane also impairs it permeability and fusibility and thus sperm membrane and oocyte penetration Previous studies have shown improvement in sperm PM, SC, decrease in seminal OS, DNA damage, levels of mutagenic base adduct(8-hydroxy-2-deoxyguanosine) in the sperm DNA^3,16–18^and improvement in the TAC (total antioxidant capacity) levels^13,16,19^ with yoga. Improvement in the quality of life by affecting the physiological and psychological counterparts of EPL and infertility has positively observed to affect the testicular functions, stabilization of the levels of reproductive hormones, semen parameters, genomic integrity, telomere length, de novo mutation rate, gene expression, and sperm epigenome^3,13,14,16–18^. The impending ROS levels correlated negatively with both PM and SC, and positively with DFI levels at baseline.

Sperm mitochondria are the major source of ROS generated due to leakage of electrons by the respiratory chain. ROS affects mitochondrial membrane potential (MMP), lipid peroxidation, sperm motility etc. Mitochondrial dysfunction results in supraphysiological production of free radicals also impairs motility due to reduced ATP production and thus fertilization is impaired. Excess ROS production by dysfunctional mitochondrial induce both denovo germ line mutations and epimutations. The improvement of mitochondrial integrity will prevent the generation and transmission of these harmful mutagenic base adducts to the embryo thus preventing any adverse effects.

Impact of yoga on OS has also been assessed by various researchers in other conditions^20–22^. Yoga has shown to aid in the amelioration of mental stress and depression by improving the feeling of perceived well-being^12,17,23–25^. Yoga has helped to normalize the levels of cortisol, improves BDNF, DHEA, serotonin which promote neuroplasticity in brain and improvement in lipid profiles, diabetic profile, and inflammatory processes^12,15,26–28^.This aids in emotional resilience and enhances metacognition.

DNA damage in our study was measured by SCSA which measures the vulnerability of sperm DNA to denaturation when exposed to heat or acid. It uses flow cytometry which, although is costly and complex but offers the analysis of large number of sperm cells (5000–10,000 spermatozoa). It has higher efficiency, objectivity, accuracy, repeatability (0.98–0.99 in clinical settings) and results are reproducible.It has advantages over other techniques like COMET and Halosperm as they are able to measure only 50–200 sperm cells per sample. Also COMET assay is very labor intensive, and halo size may vary depending o size of nuclei in Halosperm and thus may lead to variability in assessment. Both have interobserver variability as well.

DNA damage results mainly due to aberrant DNA damage response (DDR) pathway, which is an integral requirement for DNA repair and to monitor DNA integrity. Reduction of DNA damage by yoga in our study suggests the potential role of yoga in optimizing the DDR pathway and facilitating improvement in genomic integrity. Optimal ROS levels, telomere stability along with changes in mind–body communicative and neural markers by yoga contribute to genomic integrity and positive modulation of sperm epigenome^14,16,18^. Our previous studies reported a significant decline in the ROS levels by adopting yoga for 21 days^3,16^. Effect of decline in ROS with a parallel increase in TAC levels has been observed as early as 10 days^19,29,30^. But the impact of yoga to show significant improvement sperm genomic integrity (sperm DFI levels) takes longer time as the cycle of spermatogenesis is 72 days and usually significant improvement takes 2 spermatogenic cycles. The levels of DFI in our study showed an insignificant decline in this short duration and needs longer daily practice (6 months) to show significant improvement^19^.

OS is one of the predominant causes which affects the complex trait i.e. the telomeres causing telomere attrition, instability of the sperm genome, cellular senescence and testicular aging^2,17,31^.Telomeres are one of the important markers of cellular and testicular aging, which are affected by adoption of yoga^17,32,33^. Telomere length (TL) is found to be affected by OS, inflammation, various environmental, lifestyle, occupational factors, and exposure to carcinogens and various cross-sectional epidemiological studies have also been conducted^34–37^. Telomeres are rich in guanine and are thus more prone to oxidative attack. The highly truncated BER mechanism makes sperm less adept in repairing oxidative damage and results in persistent DNA damage in the telomeres leading to recruitment of DNA damage response machinery and causing telomere attrition.

Both TL and telomerase activity have been statedto show sensitivity to an array of psychosocial and behavioural influences^38–40^, thus suggesting that psychological stress is associated with reduced total antioxidant capacity and increased cortisol levels which further induces oxidative stress and is thus associated with shorter telomeres and accelerated aging. Optimal telomere length is required for normal cleavage and shorter telomeres may impair cleavage and this blastocyst development.

Only two studies have been conducted to assess the TL with time. These were conducted in cancer patients who underwent a Mindfulness Based Stress Reduction (MBSR) program for 8 weeks^41,42^. Low and high levels of OS result in shorter telomeres and genomic instability, while mild levels of OS are beneficial in maintenance of STL^43^. TL in current study was lower as compared to controls, correlated negatively with ROS and DFI, as well as showed a significant increase with yoga practice. This thus suggests that psychosocial stressors and their biochemical consequences have the potential to cause accelerated telomere erosion. Yoga practice enhances physical and emotional self-efficacy and resilience, decrease inflammation and OS and induces relaxation response. Regular daily practice is assocauted with reduced size of amygdale and increased gray matter in hippocampus and prefrontal cortex associated with improved memory, mindfulness, improved cognitionand promotes neuroplasticity and thus increases resilience.

Our study analysed the expression of 14 genes post adoption of yoga. The relative expression of the genes (average delta Ct) assessed post-yoga was found to normalize towards the levels in fertile controls. The fold change in the expression of these genes post-yoga was assessed as compared to baseline i.e. pre-yoga levels.

*FOXG1*, a key regulator of neurogenesis development of the ventral telencephalon in forebrain is responsible for early embryo patterning^44^, The expression of *FOXG1* is dose dependent^45^and had showed upregulation in RPL and RIF group post adoption of yoga with respect to pre-yoga levels. The expression of *SOX3*, a X-linked transcription factorplaying a crucial role in development of central nervous system and hypothalamus^46,47^, showed downregulation in patient groups in our study. The downregulation has previously been associated with reduced spermatogenesis and impact fertility^48^. The expression of *SOX3* is also dosage dependent and showed an upregulation in expression post-yoga in both RPL and RIF group. The expression of *STAT4* gene coding the transcription factor STAT4 showeddownregulation post-yoga in both patient groups.

The ribosomal protein coding genes *RPS6, RBM9* and *RPL10A,*associated with neurodevelopment^49^, alternative axon splicing in brain^50^and development of neural precursor cells^51^showed upregulation at baseline found to be downregulated inRPL group post-yoga. These were stated to correlate with miscarriage rate in previous studies^52,53^.RPS6, is a substrate for inducible phosphorylation, downstream effector of mTOR pathway. Hyperphosphorylation and dephosphorylation of RPS6 are associated with neurodevelopmental disorders and periods of growth arrest^49^.

The expression of *WNT5A, HSP90, TOMM7* and *EIF5A* showed upregulation in RPL and RIF groups and downregulation post-yoga. *WNT5A,* a member of WNT family plays an important role in early embryogenesis^54,55^and an altered expression might be a potential cause of pregnancy loss and implantation failure. It has also been seen to hold important role in germ cell migration and testis development^56^. It becomes pertinent here to mention the downregulation of expression of *HSP90* and *TOMM7* with yoga. HSP90 is a cytoplasmic chaperone responsible for various developmental regulatory networks^57^. HSP90 protein forms an integral part of preprotein translocase complex of the outer mitochondrial membrane (TOM complex) of TOMM7^58^. The expression of both *HSP90* and *TOMM7* showed significant correlation with ROS in RPL group, thus it can infer that reduction in ROS levels with yoga also catered to the normalization of gene expression.Eukaryotic translation initiation factor (*EIF5A*) is actively involved in cell cycle progression, mRNA decay and is involved pathways associated with stress response and maintenance of integrity of the cell wall. The overexpression of *eIF5A1*has been found to result in the loss of transmembrane potential of the mitochondrial membrane^59^.

The expression of DNA damage detection *(PARP1)* and repair *(OGG1)* genes showed upregulation post-yoga, suggesting an increase in DNA damage detection and repair activityThe expression of *PARP1* showed significant correlation with both ROS and DFI in RPL group, while*OGG1*expression significantly correlated with DFI levels. Thus, it can be inferred that the reduction in the levels of both ROS and DFI post-yoga might have facilitated the change in the gene expression and OS is major player in regulating the epigenome.

Previously, an upregulation of genes involved in cellular repair, and a downregulation of pro-inflammatory genes were observed in our patient cohort^23,60^. This evidence-based study has shown that yoga has tremendous therapeutic potential in management of cases with seminal OS, DNA damage, shorter telomeres and dysregulation of sperm transcripts. Thus, yoga may be used as an adjunct in management of such cases.

### Yoga decreases psychological stress, improves quality of life

Yoga and meditation based practices have been associated with positive physical, psychological and health benefits in research over the years. The biological mechanisms, which mediate the subjective improvements in an individual include physiological, psychological, immunological, neuroendocrine and genomic changes. Yoga enhances biological functioning by modulating the changes at the hypothalamic pituitary axis and by regulation of the inflammatory responses^12,15,61–64^.

Psychological stress, depression and anxiety are common in couples with infertility and adverse reproductive outcomes. Hypothalamo-pituitary-adrenal axis plays a pivotal role in any response to stressful stimuli^12^,^65^ and exhibits a reciprocal relationship with the hypothalamic-pituitary–gonadal (HPG) axes. Testicular tissue from rats exposed to acute stress showed higher levels of cortisol in addition to apoptosis of germ cells and Leydig cells^11^.HPA affects the synthesis and release of GnRH, LH/FSH from brain and by directly affecting gametogenesis. Glucocorticoid levels increase OS, decrease antioxidant capacity, are neurotoxic, also associated with decreased levels of BDNF and DHEA leading to depression and adversely affecting HPG and thus negatively impact reproductive health.

## Methods

### Study design and participants

**Figure 3:**
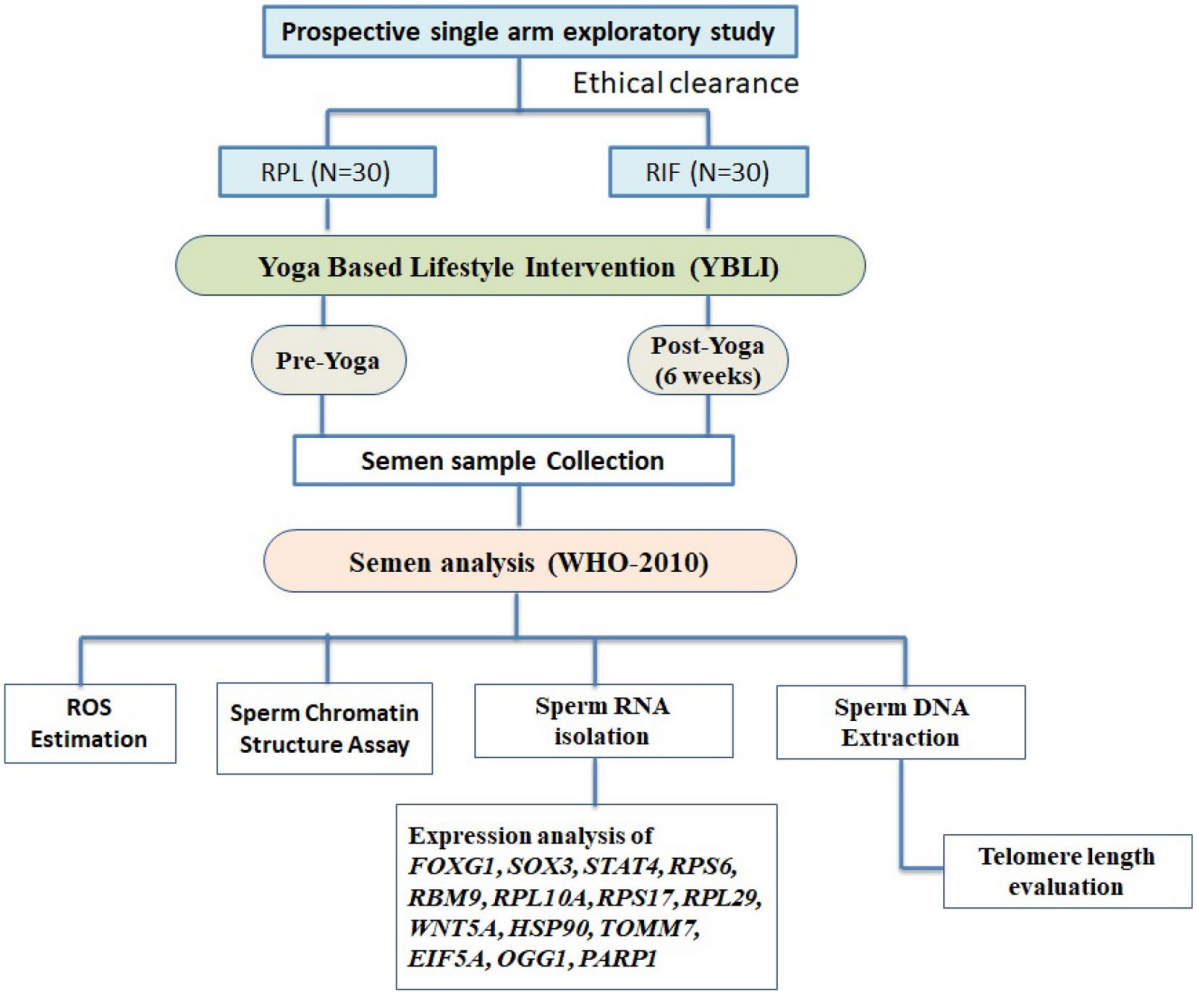
Study Design: Prospective single arm exploratory study.

The current study was a prospective single arm exploratory study (Fig. 3). The study was initiated after approval from the “Institute Ethics Committee for Post Graduate Research, All India Institute of Medical Sciences (Ref. No. IECPG-325/29-06-2016)”. The authors confirm that all experiments were performed in accordance with relevant guidelines and regulations. The patients were referred from the Department of Obstetrics and Gynecology, All India Institute of Medical Sciences, New Delhi, India.

### Participants and eligibility criteria

The participants recruited in the current study included the male partners of couples who previously experienced RPL (N = 30) and RIF (N = 30).It was a pilot study done for the first time to study the impact of yoga on the semen parameters and 30 participants were selected in each group. Semen samples were also obtained from 30 healthy fertile men who had recently fathered a child in the last two years, but they were not included for the yoga program. The patients and controls were age matched and within the age group of 18–45 years and informed written consent was obtained. Cytogenetic analysis was done in both partners to rule out any chromosomal abnormality. A complete clinical, gynecological and radiological examination as well as hormonal evaluation (T_3_, T_4_, TSH, FSH, LH, PRL) and acquired or inherited thrombophilias was conducted in the female partners to exclude any potential maternal causes of EPL.

### Yoga program

All participants were provided with a detailed description of the predesigned yoga program, which is a tested program conducted for an average 2 h per day. Keeping into consideration the overwhelming stress and anxiety associated with RPL and RIF, this integrative health strategy program design included a series of physical postures (*asanas*) which increase pelvic blood flow, breathing exercises, meditation (Table 4). These sessions were conducted for 6 days a week for an initial period of 2 weeks at the Laboratory of Molecular Reproduction & Genetics, Department of Anatomy, AIIMS, New Delhi under the able guidance and supervision of trained, registered yoga instructors.

**Table 4:** Details of practices done in a session of Yoga.

S.No	Practices done			Duration
1	Session preparation	Standing	Tadasana	5 min
2	Starting prayer			3 min
3	Loosening practices (sukshmavyayama)			5 min
4	Sun salutations (Surya Namaskar) with mool band			10 min
5	Asanas	Standing	Tadasana	20 min
			Ardhachakrasana	
			Padahastasana	
			Vrikshasana	
		Sitting	Paschimattanasana	
			Janu Sirasana	
			BadhaKanasana	
			Vakrasana	
		Prone	Bhujangasana	
			Salabhasana	
			Naukayasana	
			Makrasana	
		SUPINE	Uttanapadasana	
			Malasana	
			Pavanamukhtasana	
			Matsyasana	
6	Relaxation		Shavasana	5 min
7	Pranayama (Breathing Exercise)			20 min
			Nadishodhana	
			Bhramari	
			Kapal Bhati	
8	Dhayana		Brahmamudra	5 min
9	Nada Anusandhana (A-U-M chanting)			5 min
10	Closing prayer			7 min
11	Interactive session			15 min
12	Total			120 min

### Outcome measures

1. To evaluate the levels of seminal OS, DNA damage, telomere length and gene expression at baseline in both RPL and RIF groups and to assess the change from baseline (pre-yoga) to after 6 weeks of adopting yoga program.
2. To measure the change in quality of life (QOL) in RPL and RIF patients measured by the WHO Quality of Life BREF (WHOQOL-BREF) questionnaire.

### Assessment of study parameters

The laboratory assessment was done for various parameters at the start of yoga program (baseline, day 0) and at the end of active yoga program (after 6 weeks). Semen samples wereanalyzedas per WHO guidelines (2010). Reactive oxygen species (ROS) levels were measured by luminol-dependent chemiluminescence with luminometer (Sirius; Berthold Detection Systems GmbH, Pforzheim, Germany). The ROS values were expressed as relative light units (RLU)/sec/10^6^ spermatozoa.The DNA damage was expressed as DNA fragmentation index (DFI), measured by sperm chromatin structure assay (SCSA) as per our previously described protocol^4^. Total RNA was isolated from 1 × 10^7^ spermatozoa by TRIZOL method as described in our previous study^4^. The relative quantification of target genes was calculated with 2^-ΔΔCt^method after normalization of the amount of expressed mRNA using 2 internal housekeeping genes *β-actin* and *GAPDH*. Each cDNA product was tested in duplicate. The genes selected in the study were based on a previous microarray analysis done in our laboratory and were also selected from the previous reported literature where they affect fertilization, implantation and pregnancy success.

Sperm telomere length was determined from the sperm DNA by a quantitative real-time PCR-based method^34^. Briefly, the relative mean telomere length was determined by comparing the value from absolute quantification of telomere DNA with a single copy reference gene, 36B4 (T/S ratio).

The assessment of QOL was made by the WHOQOL-BREF questionnaire which contains 26 original items, including 2-items examining an individual’s overall perception of the QOL and health; 24 items examining 4-domains (D1, physical; D2, psychological; D3, social; and D4, environmental)^66^. The questionnaire depicts a score ranging from 0 to 100, in which a higher score denotes a better QOL.

### Statistical analysis

Data was analysed by statistical software Stata 14.0. Categorical data expressed as frequency and percentage. Quantitative data was expressed as mean ± SD and median (min–max) followed normal and skewed distribution respectively. The changes within group over time (pre- to post-yoga) were assessed using paired t-tests for normally distributed continuous variables, or Wilcoxon signed rank test for continuous variables without normal distribution. Pearson and Spearman correlation coefficient was used to find the relationship between selected markers as appropriate.Significance was accepted at *p* < 0.05.

### Ethical approval

The study was initiated after approval from the “Institute Ethics Committee for Post Graduate Research, All India Institute of Medical Sciences (Ref. No. IECPG-325/29-06-2016)”. The authors confirm that all experiments were performed in accordance with relevant guidelines and regulations.

### Consent to participate

Informed consent was obtained from all individual participants included in the study.

### Consent to publish

The manuscript doesn’t contain any individual details, images or videos of the patients. Consent to publish the data had been obtained.

## Limitations and strengths

Limitations in the study included small sample size and short duration 6 weeks to ensure better compliance, inclusion of participants from Indian nationality.Yoga should be practiced daily and in these studies major limitation is drop out rate and one hour daily practice To the best of our knowledge this is the first study to assess the impact of adoption of yoga on the seminal biomarkers, telomere length, DNA integrity and gene expression in EPL.

## Summary and conclusion

Sperm is a polarised cell highly vulnerable to environmental insults as it accumulates DNA damage due to a highly truncated reapir mechanism and minimal cytosolic antioxidants. Sperm factors role extends beyond fertilization with restoration of diploidy but sperm are critical determinants of embryo viability and developmental competence. Oxidative stress, oxidative DNA damage, short telomere length and the dysregulated gene expression might adversely affect pregnancy outcomes. Pregnancy loss is associated with comorbid stress, anxiety and depression. Yoga is a mind body medicine and results in a significant improvement of both mental and physical health and improvement in all domains of quality of life. The decrease in the ODD prevents *denovo* germ line mutations and epimutations. Regular Yoga practice with improvement in sperm parameters and improvement in both nuclear and mitochondrial DNA integrity may aid in couples to conceive spontaneously and also improve success rates after IVF/ICSI by reducing incidence of pregnancy losses, implantation failures and significant morbidity and mortality in the offspring.

## Data Availability

The datasets generated during and/or analysed during the current study are available from the corresponding author on reasonable request.

## References

[1] Aitken RJ, Drevet JR (2020). The importance of oxidative stress in determining the functionality of mammalian spermatozoa: A two-edged sword. Antioxidants (Basel, Switzerland).

[2] Dhawan V, Kumar M, Dada R (2017). Effect of sperm molecular factors, oxidative damage and transcripts in childhood disorders. J. Child Dev. Disord.

[3] Dhawan V (2018). Meditation & yoga: Impact on oxidative DNA damage & dysregulated sperm transcripts in male partners of couples with recurrent pregnancy loss. Indian J. Med. Res..

[4] Dhawan V (2019). Paternal factors and embryonic development: Role in recurrent pregnancy loss. Andrologia..

[5] Eisenberg ML, Li S, Cullen MR, Baker LC (2016). Increased risk of incident chronic medical conditions in infertile men: analysis of United States claims data. FertilSteril.

[6] Latif T (2017). Semen quality as a predictor of subsequent morbidity: A Danish Cohort Study of 4712 men with long-term follow-up. Am. J. Epidemiol..

[7] Eisenberg ML (2014). Semen quality, infertility and mortality in the USA. Hum Reprod..

[8] Jensen TK, Jacobsen R, Christensen K, Nielsen NC, Bostofte E (2009). Good semen quality and life expectancy: A cohort study of 43,277 men. Am. J. Epidemiol..

[9] Gollenberg AL (2010). Semen quality in fertile men in relation to psychosocial stress. Fertil. Steril..

[10] Corona G (2016). Testosterone supplementation and body composition: results from a meta-analysis study. Eur. J. Endocrinol..

[11] Ilacqua A, Izzo G, Emerenziani GP, Baldari C, Aversa A (2018). Lifestyle and fertility: the influence of stress and quality of life on male fertility. Reprod. Biol. Endocrinol..

[12] Tolahunase MR, Sagar R, Faiq M, Dada R (2018). Yoga- and meditation-based lifestyle intervention increases neuroplasticity and reduces severity of major depressive disorder: A randomized controlled trial. Rest. Neurol. Neurosci..

[13] Dhawan V, Dada R, Parekattil SJ (2020). Yoga, meditation and acupuncture for male reproductive health. Male Infertility.

[14] Dhawan V, Kumar M, Chaurasia P, Dada R (2019). Mind-body interventions significantly decrease oxidative DNA damage to sperm genome: clinical implications. ROS..

[15] Gautam S, Tolahunase M, Kumar U, Dada R (2019). Impact of yoga based mind-body intervention on systemic inflammatory markers and co-morbid depression in active Rheumatoid arthritis patients: A randomized controlled trial. Restor. Neurol. Neurosci..

[16] Dhawan V, Kumar R, Malhotra N, Singh N, Dada R (2018). Yoga based lifestyle intervention in the management of recurrent implantation failure. Ind. J. Sci. Res..

[17] Dada R, Kumar SB, Tolahunase M, Mishra S, Mohanty K, Mukesh T (2015). Yoga and meditation as a therapeutic intervention in oxidative stress and oxidative DNA damage to paternal genome. J. Yoga Phys. Ther..

[18] Rima D, Shiv BK, Bhawna Ch, Shilpa B, Saima Kh (2016). Oxidative stress induced damage to paternal genome and impact of meditation and yoga- can it reduce incidence of childhood cancer?. Asian Pac. J. Cancer Prev..

[19] Kumar SB (2015). Improvement in sperm DNA quality following simple lifestyle intervention: A study in fathers of children with non-familial sporadic heritable retinoblastoma. J. Clin. Case Reports.

[20] Gordon L, McGrowder DA, Pena YT, Cabrera E, Lawrence-Wright MB (2013). Effect of yoga exercise therapy on oxidative stress indicators with end-stage renal disease on hemodialysis. Int. J. Yoga..

[21] Patil SG, Dhanakshirur GB, Aithala MR, Naregal G, Das KK (2014). Effect of yoga on oxidative stress in elderly with grade-I hypertension: A randomized controlled study. J. Clin. Diag. Res..

[22] Cheung C (2018). Effects of yoga on oxidative stress, motor function, and non-motor symptoms in Parkinson's disease: a pilot randomized controlled trial. Pilot Feasibility Stud..

[23] Dada T (2018). Mindfulness meditation reduces intraocular pressure, lowers stress biomarkers and modulates gene expression in glaucoma: A randomized controlled trial. J. Glaucoma.

[24] Dada T, Yadav RK, Faiq MA (2018). Effect of yoga-based ocular exercises in lowering of intraocular pressure in glaucoma patients: An affirmative proposition. Int. J. Yoga..

[25] Hagins M, States R, Selfe T, Innes K (2013). Effectiveness of yoga for hypertension: systematic review and meta-analysis. Evid. Based Complement. Alternat. Med..

[26] Gagrani M (2018). Meditation enhances brain oxygenation, upregulates BDNF and improves quality of life in patients with primary open angle glaucoma: A randomized controlled trial. Restor. Neurol. Neurosci..

[27] Raveendran AV, Deshpandae A, Joshi SR (2018). Therapeutic role of yoga in type 2 diabetes. Endocrino. lMetab. (Seoul).

[28] Aschbacher K, O'Donovan A, Wolkowitz OM, Dhabhar FS, Su Y, Epel E (2013). Good stress, bad stress and oxidative stress: Insights from anticipatory cortisol reactivity. Psychoneuroendocrinol..

[29] Sharma H, Sen S, Singh A, Bhardwaj NK, Kochupillai V, Singh N (2003). Sudarshan Kriya practitioners exhibit better antioxidant status and lower blood lactate levels. Biological Psychol..

[30] Sinha S, Singh SN, Monga YP, Ray US (2007). Improvement of glutathione and total antioxidant status with yoga. J. Altern. Complement. Med..

[31] Fouquerel E (2016). Oxidative guanine base damage regulates human telomerase activity. Nat. Struct. Mol. Biol..

[32] Hoge EA (2013). Loving-kindness meditation practice associated with longer telomeres in women. Brain Behav. Immun..

[33] Alda M (2016). Zen meditation, length of telomeres, and the role of experiential avoidance and compassion. Mindfulness.

[34] Thilagavathi J (2013). Analysis of telomere length in couples experiencing idiopathic recurrent pregnancy loss. J. Assist. Reprod. Genet..

[35] Thilagavathi J, Kumar M, Mishra SS, Venkatesh S, Kumar R, Dada R (2013). Analysis of sperm telomere length in men with idiopathic infertility. Arch. Gynecol. Obstet..

[36] Starkweather AR (2014). An integrative review of factors associated with telomere length and implications for biobehavioral research. Nurs. Res..

[37] Patel CJ, Manrai AK, Corona E, Kohane IS (2017). Systematic correlation of environmental exposure and physiological and self-reported behaviour factors with leukocyte telomere length. Int. J. Epidemiol..

[38] Blackburn EH, Epel ES, Lin J (2015). Human telomere biology: A contributory and interactive factor in aging, disease risks, and protection. Science.

[39] Puterman E, Epel E (2012). An intricate dance: Life experience, multisystem resiliency, and rate of telomere decline throughout the lifespan. Soc. Pers. Psychol. Compass.

[40] Shalev I (2013). Stress and telomere biology: A lifespan perspective. Psychoneuroendocrinol..

[41] Lengacher CA (2014). Influence of mindfulness-based stress reduction (MBSR) on telomerase activity in women with breast cancer (BC). Biol. Res. Nurs..

[42] Carlson LE (2015). Mindfulness-based cancer recovery and supportive–expressive therapy maintain telomere length relative to controls in distressed breast cancer survivors. Cancer.

[43] Misra S, Kumar R, Malhotra N, Singh N, Dada R (2016). Mild oxidative stress is beneficial for sperm telomere length maintenance. World J. Methodol..

[44] Tian C, Gong Y, Yang Y, Shen W, Wang K, Liu J, Xu B, Zhao J, Zhao C (2012). Foxg1 has an essential role in postnatal development of the dentate gyrus. J. Neurosci..

[45] Hettige NC, Ernst C (2019). *FOXG1* dose in brain development. Front. Pediatr..

[46] Bergsland M, Ramsköld D, Zaouter C, Klum S, Sandberg R, Muhr J (2011). Sequentially acting Sox transcription factors in neural lineage development. Genes Dev..

[47] Topalovic V (2017). Epigenetic regulation of human SOX3 gene expression during early phases of neural differentiation of NT2/D1 cells. PLoS One.

[48] Raverot G, Weiss J, Park SY, Hurley L, Jameson L (2005). Sox3 expression in undifferentiated spermatogonia is required for the progression of spermatogenesis. Develop. Biol..

[49] Chen B, Tan Z, Gao J, Wu W, Li L (2015). Hyperphosphorylation of ribosomal protein S6 predicts unfavorable clinical survival in non-small cell lung cancer. J. Exp. Clin. Cancer Res..

[50] Guallar D, Wang J (2014). RNA-binding proteins in pluripotency, differentiation and reprogramming. Front. Biol..

[51] Chau KF (2018). Downregulation of ribosome biogenesis during early forebrain development. Elife..

[52] Bonache S, Mata A, Ramos MD, Bassas L, Larriba S (2012). Sperm gene expression profile is related to pregnancy rate after insemination and is predictive of low fecundity in normozoospermic men. Hum. Reprod..

[53] Ferlin A (2012). New genetic markers of male infertility. Asian J. Androl..

[54] Maeda K (2018). Wnt5a-Ror2 signaling between osteoblast-lineage cells and osteoclast precursors enhances osteoclastogenesis. Nat. Med..

[55] Van Amerongen R, Fuerer C, Mizutani M, Nusse R (2012). Wnt5a can both activate and repress Wnt/β-catenin signaling during mouse embryonic development. Dev. Biol..

[56] Tevosian SG (2012). Gone without the WNT: A requirement for WNT5A in germ cell migration and testis development. Biol. Reprod..

[57] Condelli V (2019). HSP90 molecular chaperones, metabolic rewiring, and epigenetics: Impact on tumor progression and perspective for anticancer therapy. Cells..

[58] Reyes A, Melchionda L, Burlina A, Robinson AJ, Ghezzi D, Zeviani M (2018). Mutations in TIMM50 compromise cell survival in OxPhos-dependent metabolic conditions. EMBO Mol. Med..

[59] Sun Z, Cheng Z, Taylor CA, McConkey BJ, Thompson JE (2010). Apoptosis induction by eIF5A1 involves activation of the intrinsic mitochondrial pathway. J. Cell Physiol..

[60] Dada, T. et al. Effect of yoga and meditation based intervention on intraocular pressure, quality of life, oxidative stress and gene expression pattern in primary open angle glaucoma: ARandomized Controlled Trial. *Investigat. Ophthal. Vis Sci*. **57** (2016).

[61] Dahl CJ, Lutz A, Davidson RJ (2015). Reconstructing and deconstructing the self: cognitive mechanisms in meditation practice. Trends Cogn. Sci..

[62] Tang Y, Holzel BK, Posner MI (2015). The neuroscience of mindfulness meditation. Nat. Rev. Neurosci..

[63] Luders E, Cherbuin N (2016). Searching for the philosopher’s stone: Promising links between meditation and brain preservation. Ann. N. Y. Acad. Sci..

[64] Whirledge S, Cidlowski JA (2010). Glucocorticoids, stress, and fertility. Minerva Endocrinol..

[65] Chen KW (2012). Meditative therapies for reducing anxiety: a systematic review and meta-analysis of randomized controlled trials. Depress. Anxiety.

[66] Gholami A, Jahromi LM, Zarei E, Dehghan A (2013). Application of WHOQOL-BREF in measuring quality of life in health-care staff. Int. J. Prev. Med..

